# Evidence of insecticide resistance selection in wild *Anopheles coluzzii* mosquitoes due to agricultural pesticide use

**DOI:** 10.1186/s40249-019-0572-2

**Published:** 2019-07-15

**Authors:** Chouaïbou Seïdou Mouhamadou, Sarah Souline de Souza, Behi Kouadio Fodjo, Marius Gonse Zoh, Nestor Kesse Bli, Benjamin Guibehi Koudou

**Affiliations:** 1 Centre Suisse de Recherches Scientifiques en Côte d’Ivoire (CSRS), Abidjan, Côte d’Ivoire; 2 Department of Entomology and Plant Pathology, North Carolina State University, Raleigh, NC USA; 3 Bayer West-Central Africa SA, Abidjan, Côte d’Ivoire; 4 Université Nangui Abrogoua, Abidjan, Côte d’Ivoire; 5 Laboratoire d’Ecologie Alpine (LECA), CNRS, Grenoble, France; 6 Université Grenoble-Alpes, Grenoble, France; 7 Environmental and Systems Biology (BEeSy), Université Grenoble-Alpes, Grenoble, France

**Keywords:** Vector control, Insecticide resistance, Resistance selection, Agriculture, *Anopheles coluzzii*

## Abstract

**Background:**

The wetlands used for some agricultural activities constitute productive breeding sites for many mosquito species. Thus, the agricultural use of insecticide targeting other pests may select for insecticide resistance in malaria mosquitoes. The purpose of this study is to clarify some knowledge gaps on the role of agrochemicals in the development of insecticide resistance in malaria vectors is of utmost importance for vector control.

**Methods:**

Using the CDC bottle test and the log-probit analysis, we investigated for the first time the resistance levels of *Anopheles coluzzii* mosquitoes to neonicotinoids, insecticides used exclusively for crop protection in Côte d’Ivoire. The study was conducted in two agricultural regions (Tiassale and Gagnoa) and one non-agricultural region (Vitre) between June and August 2017 using clothianidin, acetamiprid and imidacloprid.

**Results:**

Mosquito populations from Tiassale and Gagnoa (agricultural settings) were determined to be resistant to acetamiprid with mortality rates being < 85% at 24 h post-exposure. In Vitre (non-agricultural area) however, the mosquito population was susceptible to acetamiprid. In all three localities, mosquito populations were resistant to imidacloprid (mortality rates were 60% in Vitre, 37% in Tiassale, and 13% in Gagnoa) and completely susceptible to clothianidin (100% mortality). *An. coluzzii* represented 100% of mosquito collected in Gagnoa, 86% in Tiassale and 96% in Vitre.

**Conclusions:**

This study provides strong evidence that agricultural use of insecticides can cause insecticide resistance in malaria vector populations. Insecticide resistance driven by agrochemical usage should be considered when vector control strategies are developed.

**Electronic supplementary material:**

The online version of this article (10.1186/s40249-019-0572-2) contains supplementary material, which is available to authorized users.

## Multilingual abstracts

Please see Additional file 1 for translations of the abstract into the five official working languages of the United Nations.

## Background

The precipitous rise in insecticide resistance among disease vectors makes the development of new insecticides for vector control important now more than ever. There are a range of insecticide classes used in agriculture that have not yet been applied to public health, such as the neonicotinoids. Some of these likely will provide additional modes of action for insecticide resistance management, particularly until new modes of action dedicated to vector control become available.

The neonicotinoid family is composed of eight active ingredients which includes imidacloprid, thiamethoxam, thiacloprid, nitenpyram, acetamiprid, clothianidin, dinotefuran and nithiazine. These have a unique mode of action from other insecticides currently used in public health, hence their potential value in resistance management. They act by selectively targeting the invertebrate nicotinic acetylcholine receptor (nAChR) and disrupting excitatory cholinergic neurotransmission leading to paralysis and death [1]. Neonicotinoids are widely used in agriculture. Their pest spectrum, systemic activity and relatively low risk to non-target organisms have resulted in their widespread use. They constituted more than 25% of the insecticides sold globally in 2014 [2]. Imidacloprid, the first neonicotinoid developed for commercial use, was introduced in 1991. While it is currently the most widely-used neonicotinoid, several others have been developed and implemented since its inception.

Interest in neonicotinoids for vector control is focused around clothianidin, which was developed by both Sumitomo (solo, under the brand name SumiShield) and Bayer (as a combination with deltamethrin, under the brand name Fludora Fusion). These lead to a need to better understand the current susceptibility profiles of *Anopheles* spp. malaria mosquitoes to this family of compounds, especially in areas where agricultural usage is high. Several studies have proposed the link between agricultural pesticide use and the development of insecticide resistance in malaria vectors [3–8], although direct causal links have been difficult to establish, as these studies focused on insecticide modes of action that are used both in agriculture and in public health.

In the study described herein, neonicotinoids were never involved in any vector control strategy in Côte d’Ivoire, so any potential resistance observed in *Anopheles* spp. vectors to these products could only be attributed to their use in agriculture. Three neonicotinoids were assessed in this study: clothianidin, acetamiprid and imidacloprid. We sought to evaluate, for the first time, the resistance level of natural malaria vectors to neonicotinoids in different agrochemical use contexts, and to generate data that could serve as a basis for discussion of novel neonicotinoid-based vector control strategies. Addressing these also will clarify important knowledge gaps on the role of agrochemicals in the development of insecticide resistance in malaria vectors and implications for malaria vector control interventions.

## Methods

### Study sites

The study was conducted at three different geographical areas: Vitre (5°15′44“ N, 3°45’14” W), Tiassale (5°53′54“ N, 4°49’42” W) and Gagnoa (6°08′00″ N, 5°56′00″ W) in Côte d’Ivoire, that varied by agricultural profile (i.e. the main crops grown in that area) and the subsequent use of agrochemicals. Vitre is a peri-urban area located 30 km to the southeast of Abidjan, the economic capital of Côte d’Ivoire. Its climate is tropical, with a short dry season, and a rainy season marked by high rainfall most months of the year (average precipitation of 1912 mm of rain) and an average temperature of 26.5 °C. Food production, which is the main agricultural activity of the region is not as practiced as lagoon fishing which constitutes the main economic activity of residents. Gagnoa is located approximately at 270 km northeast of Abidjan. The prevailing climate is humid tropical and has four seasons including a long rainy season followed by a short dry season, and then a short rainy season followed by a long dry season. Temperatures range from 21 to 35 °C during the year. Gagnoa is located in a densely forested area, which is now severely degraded by the ubiquitous cacao crop; together with cassava constitutes the main economic activity of the region. Other agricultural activities include growing maize, yams, rice, coffee, plantain and other food products. A pervasive use of pyrethroids and neonicotinoids has been recorded in this region in crop protection (primarily against cocoa pests). These two insecticide classes account for more than 90% of all insecticides used in the region [9]. Tiassale is located between Abidjan and Gagnoa in southern Côte d’Ivoire, about 120 km northwest of Abidjan. The climate is tropical and also characterized by four seasons. A long rainy season during which falls 2/3 of the annual rainfall, a short dry season, then a short rainy season followed by a long dry season. Tiassale is a rice-growing irrigated area with an intensive use of agrochemicals throughout the year including pyrethroids accounting for approximately 85%, and neonicotinoids accounting for approximately 9% of all agrochemicals used [4].

### Field sampling

The study was conducted between June and August 2017 corresponding to the rainy season favourable to breeding site creation. Mosquito larvae were sampled from multiple breeding sites at each locality and pooled together, then re-distributed evenly in development trays containing fresh water. They were provided access to powdered TetraFin® fish food, and were reared to adults under insectary conditions of 25–28 °C and 70–80% relative humidity (RH) at the *Centre Suisse de Recherches Scientifiques en Cote d‘Ivoire*. Adults were maintained in 35 cm^3^ cages and allowed access to 10% sugary water.

### CDC bottle bioassays

#### Determination of neonicotinoids diagnostic doses

The determination of a diagnostic concentration was made using bottle bioassays. The treatment of bottles was conducted in compliance with US Center for Disease Control (CDC) guidelines [10, 11]. Acetamiprid used was formulated as Optimal 20SP whereas imidacloprid and clothianidin were technical materials. These chemicals were obtained from Bayer CropScience Ltd. Each chemical was mixed in acetone (or acetone and Mero for clothianidin) at different concentrations ranging from 50 to 200 μg/bottle and used for testing against the susceptible *An. gambiae* Kisumu mosquitoes to determine the diagnostic concentration. The diagnostic concentration was defined as the minimum concentration that killed 100% of susceptible mosquitoes after 0.5–1 h exposure and a 24 h holding period.

#### Assessment of the insecticide resistance level

The bioassays were performed with bottles in an upright position according to CDC guidelines [10] using 20 to 25 non-blood-fed, wild adult female *An. gambiae* sensu *lato* mosquitoes, 3 to 5 days old. Four replicates were tested per condition (Additional files 2, 3 and 4). During the exposure period, knocked down mosquitoes (mosquito lies on its back, mosquito that cannot stand, mosquito that cannot fly in coordinate manner and mosquito that can take off briefly but falls down immediately) were counted every 5 min (Additional files 2, 3 and 4). After the corresponding exposure time defined with the susceptible strain, mosquitoes were removed from the bottles and placed into net-covered plastic cups containing a 10% honey solution. They were observed for mortality daily for five consecutive days (Additional files 2, 3 and 4). Testing was performed at 25–27 °C and 70–90% RH.

### Molecular identification of mosquitoes

#### DNA extraction

A total of 50 adult mosquitoes from the negative control batches were processed for molecular identification. Genomic DNA of whole mosquitoes was extracted according to Collins et al. [12]. In brief, whole mosquitoes previously soaked individually in 200 μl of 2% cetyl trimethyl were crushed and incubated at 65 °C for 5 min. Then, 200 μl of chloroform was added and the resulting mixture was centrifuged for 5 min at 12000 rpm. The supernatant was pipetted into a new 1.5 ml tube to which 200 μl isopropanol was added; DNA was precipitated by spinning the mixture for 15 min at 12000 rpm. The supernatant was removed and the pellet of DNA formed at the bottom of tubes was purified with 70% ethanol. A further centrifugation step at 12000 rpm for 5 min was used to rinse the DNA, the excess ethanol was removed, and the resulting pellet was dried on the lab bench overnight. The extracted DNA was reconstituted in 20 μl DNase-free water (Sigma-Aldrich, United Kingdom) prior to storage at − 20 °C.

#### Identification of *Anopheles gambiae* complex members

Specimens were identified to species by Sine polymerase chain reaction (PCR) [13]. PCR reactions were carried out in a 25 μl reaction which contained 1 pmol of each of the following primer: F6.1A of sequence 5′-TCGCCTTAGACCTTGCGTTA-3′ used to determine *An. coluzzii* formerly known as *An. gambiae* M molecular form and the R6.1B of sequence 5′-CGCTTCAAGAATTCGAGATAC-3′ for *An. gambiae* formerly known as *An. gambiae* S molecular form. The other reagents included 0.2 mmol/L of each dNTP, 1.5 mmol/L MgCl_2_, 2.5 U *Taq* polymerase, and 1 μl of DNA template extracted from individual mosquitoes. The thermocycler program was: 94 °C for 5 min followed by 35 cycles of 94 °C for 25 s, 54 °C for 30 s and 72 °C for 1 min, a final elongation step at 72 °C for 10 min, and a 4 °C hold. The resulting products were allowed to migrate on a 1.5% agarose gels stained with ethidium bromide. The profile of the expected bands by species was 479 bp for *An. coluzzii* and 249 bp for *An. gambiae* s.s. after visualization with an ultra violet illuminator.

### Test data interpretation

Test data were interpreted based on World Health Organization (WHO) criteria [14], stating that: mortality < 90% is indicative of resistance, mortality levels from 90 to 97% is suggestive of probable resistance and needs further investigation, and mortality ≥98% is indicative of susceptibility. The mortality of a test sample was calculated by summing the number of dead mosquitoes across all four exposure bottles and expressing this as a percentage of the total number of exposed mosquitoes. The Abbott formula [15] was used to correct test mortality if mortality in the control was 5–20%. The test was repeated if mortality in the control was more than 20%. The time necessary to allow 50% of test mosquito populations to be knocked down (KDT_50_) was determined using the PoloPlus software 1.0 (Leora Software Services, Northampton Business Center, Northampton, UK).

via log-probit analysis, and the Resistance Ratio (RR) calculated as the KDT_50_ of the wild strain divided by the KDT_50_ of the susceptible Kisumu strain.

## Results

### Diagnostic doses

It appeared that 50 μg/bottle of clothianidin, 75 μg/bottle of acetamiprid, and 200 μg/bottle of imidacloprid were the minimum concentrations that caused 100% mortality on the susceptible Kisumu strain at 24 h post-exposure after a minimum of 30 min exposure for clothianidin and 1 h exposure for both acetamiprid and imidacloprid in CDC bottles. They were therefore considered as diagnostic concentrations for the entirety of the study.

### Knockdown and resistance ratio

**Fig. 1:**
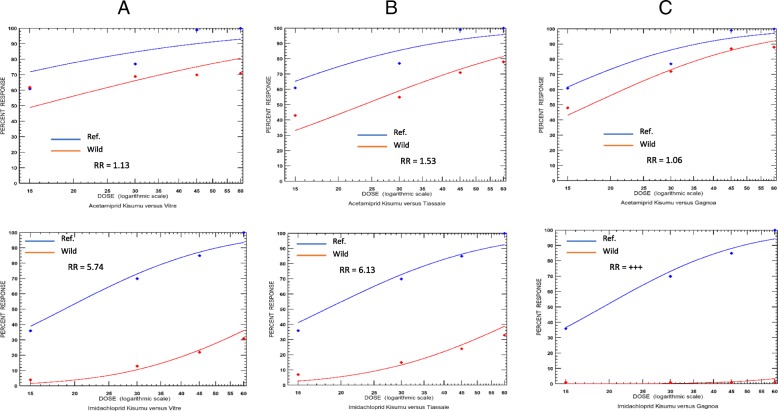
Knockdown and resistance factor of wild *Anopheles coluzzii* populations from non-agricultural (Vitre in column **a**) and agricultural areas (Tiassale in column **b** and Gagnoa in Column **c**) and exposed to three neonicotinoids. We were unable to generate a regression curve for clothianidin as only two time-points knockdown data were recorded after 30 min exposure with more than 90% mosquito’s dead in less than 15 min. The resistance ratio (RR) was calculated as the time necessary to allow 50% of test mosquitoes to be knocked down (KDT_50_) of the wild strain divided by the KDT_50_ the susceptible Kisumu strain. We could not calculate the RR for imidachloprid in Gagnoa because only one mosquito was knocked down after exposure. This was referred here as +++ (extremely high)

Knocked down mosquitoes were recorded for both acetamiprid and imidacloprid insecticides tested in the three localities, which rendered possible the determination of Resistance Ratios (RR), (Table 1). With regard to clothianidin, we were unable to determine the time needed for 50% of the population to be knocked down, as more than 90% mosquitoes dead in less than 15 min exposure to this chemical and did not allow generation of sufficient data points for a regression curve. For acetamiprid, the TKD_50_ of the Kisumu susceptible strain was 13.74 (95% *CI*: 10.47–16.08) min. This time has slightly increased among the wild mosquito populations. However, the RR remained at < 2 for the three locations (Fig. 1a). As for imidacloprid, the TKD_50_, which was 18.25 (95% *CI*: 14.94–21.88) min in the Kisumu strain, increased to 104.6 (95% *CI*: 77.2–197.7) min among the non-agricultural area of Vitre mosquito populations, and 111.6 (95% *CI*: 78.3–247.4) min in the rice cultivation area of Tiassale. Only one mosquito was knock down at the cocoa area of Gagnoa. This resulted in an RR varying from 5.747 for Vitre to 6.132 in Tiassale and indefinite in Gagnoa (Fig. 1b).

**Table 1:** Resistance ratio of wild *Anopheles coluzzii* populations from non-agricultural (Vitre) and agricultural areas (Tiassale and Gagnoa) exposed to three neonicotinoids

Localities	Insecticides	KDT50 (95% *CI*) in minutes		Resistance ratio*
		Kisumu	Wild strains	
Vitre	Acetamiprid	13.7 (10.47–16.68)	15.52 (13.6–18.013)	1.130
	Imidachloprid	18.2 (14.95–21.88)	104.6 (77.2–197.7)	5.747
Tiassale	Acetamiprid	13.7 (10.47–16.68)	20.9 (14.14–25.7)	1.526
	Imidachloprid	18.2 (14.95–21.88)	111.6 (78.3–247.4)	6.132
Gagnoa	Acetamiprid	13.7 (10.47–16.68)	15.7 (11.6–19.07)	1.062
	Imidachloprid	18.2 (14.95–21.88)	1 knock down only after 1 h	+++

KDT_50_: The time necessary to allow 50% of test mosquito populations to be knocked down. Determined using the PoloPlus software 1.0 via log-probit analysis

*Resistance ratio (RR) calculated as the KDT_50_ of the wild strain divided by the KDT_50_ of the susceptible Kisumu strain

+++ could not be calculated, indefinite

### Mortality

**Fig. 2:**
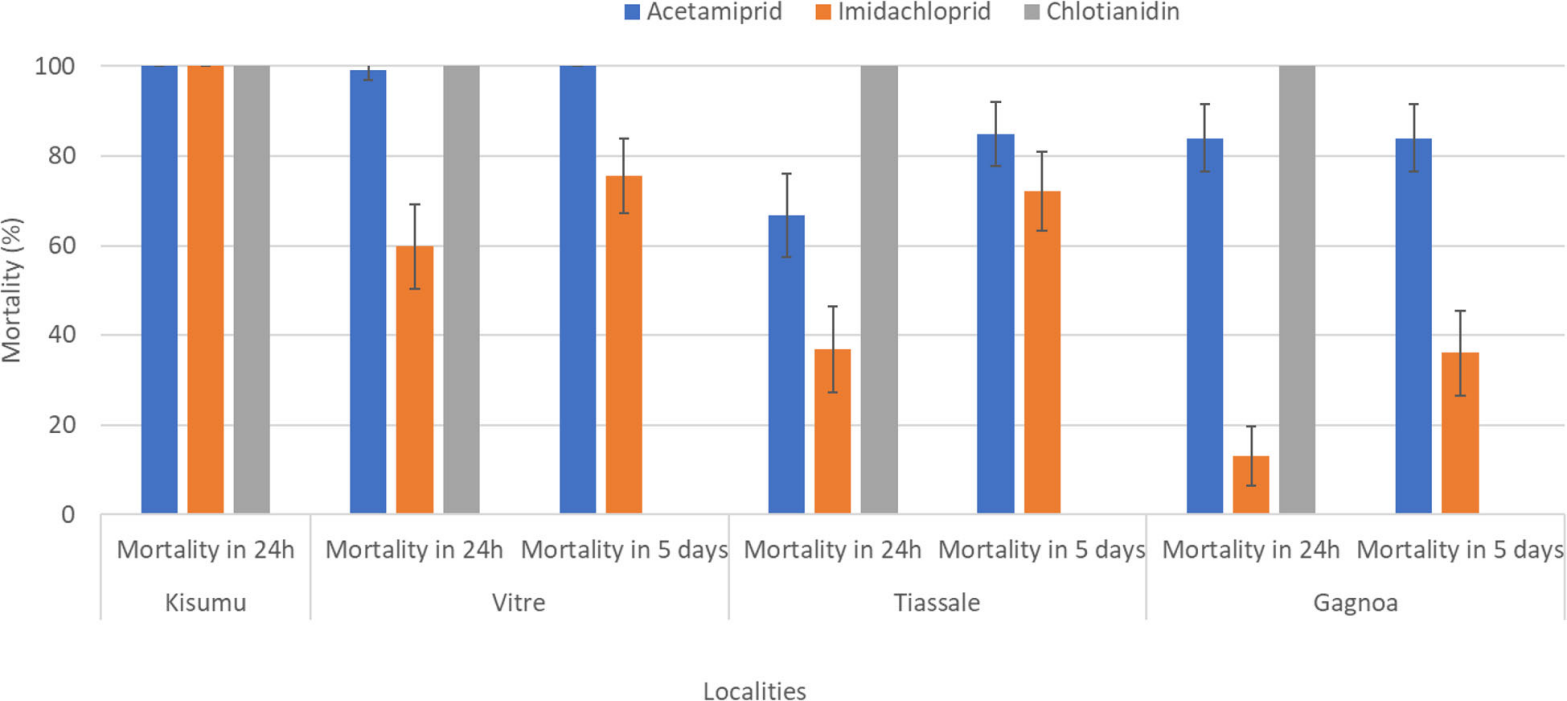
Mortality in CDC bottle bioassay of wild *Anopheles coluzzii* exposed to acetamiprid, imidachloprid and clothianidin. Vitre is a sub-urban area with a very low agricultural practice. Tiassale is a rice-growing irrigated area with intensive use of chemicals including neonicotinoids. Gagnoa on is a cocoa growing area where neonicotinoids use is common. Resistance to imidachloprid is found in all the three localities whereas resistance to acetamiprid is seen in agricultural growing areas only. No resistance was found to clothianidin. The bars represent the confidence intervals at 95% (95% *CI*)

The mosquito populations from the three localities were completely susceptible to clothianidin, as there was 100% mortality at 24 h after 30 min of exposure (Fig. 2). The mosquito populations from Tiassale and Gagnoa appeared to be resistant to acetamiprid with mortalities less than 85% even 5 days post-exposure. In Vitre however, mosquitoes displayed 99% mortality after 24 h post exposure thus reflecting complete susceptibility (Fig. 2). Concerning imidacloprid, the mosquito populations were resistant to this compound in the three localities (Fig. 2). The highest mortality was recorded in Vitre followed by Tiassale, and the lowest observed in Gagnoa. Mortalities after 24 h post-exposure decreased from 60% in Vitre, non-agricultural area, to 37% in Tiassale and 13% in Gagnoa, both agricultural settings. After 5 days, mortalities at the three localities were still less than 75%.

### Species identification

A total of 50 female mosquitoes were characterized per locality for species identification. It appeared that all individuals from Gagnoa were identified as *An. coluzzii* (100%), in Tiassale 86% of the mosquitoes were *An. coluzzii* (43 individuals were *An. coluzzii* and six were *An. gambiae*) and in Vitre 96% of the mosquitoes were *An. coluzzii* (48 individuals) and 4% (two individuals) were hybrids of *An. coluzzii* and *An. gambiae*.

## Discussion

The present study explores for the first-time resistance to neonicotinoids in the principal malaria vector *An. gambiae*. It raises the question of the importance of the impact of insecticides used in agriculture on the development of resistance in insects of public health interest. Indeed, the neonicotinoids are compounds exclusively used in agriculture. In Côte d’Ivoire, imidacloprid and acetamiprid are widely used for cocoa crop protection [9], and to a considerable extent in the protection of rice fields against pests [4]. The fact that they have never been used in public health suggests that they may have triggered the observed resistance in *Anopheles*. According to Lines [16], for an insecticide used in agriculture to be the source of insecticide resistance selection in public health insects, resistance to this compound should be observed before its use for vector control and the level of resistance should be high in areas where this compound is used compared to areas where only vector-based treatments are available. However, multiple-resistance, which encompasses neonicotinoids and other agrochemicals, is also a possibility as these insecticides are used in a program that includes applications of herbicides and fungicides. The subsequent exposure of mosquito populations to multiple biocides might select for several metabolic pathways with a putative side effect on tolerance to a broader range of insecticides, including neonicotinoids. In other insects, resistance to neonicotinoids involves either a modification of the insecticide target site, preventing the insecticide from reaching its site of action following a gene polymorphism, or an increase in the degradation of the insecticide by metabolic enzymes. The neonicotinoid insecticides kill by disrupting the normal physiological workings of the nicotinic acetylcholine receptor (nAChR), which is a ligand-gated ion channel responsible for mediating excitatory cholinergic neurotransmission in the central nervous system of invertebrates [1]. The first incidences of resistance to neonicotinoids appeared 5 years after their introduction around 1996, in a worst case scenario in glasshouse production systems where multiple insecticide applications occurred within a finite pest population that also had a high reproductive rate. Today, resistance to these products is present in a substantial number of pests and the Arthropod Pesticide Resistance Database lists more than 500 cases of resistance to neonicotinoids [17].

Mutations on the nAChR responsible for neonicotinoid resistance appear to be very rare in nature [2], but when individuals are subjected to extensive selection pressures under laboratory conditions, mutations on these receptors associated with resistance are observed. Cloning of nAChR subunits of *Nilaparvata lugens*, the brown planthopper, after selection pressure with imidacloprid over 35 generations in laboratory conditions revealed that a replacement of tyrosine with a serine was associated with a 250-fold resistance level to imidacloprid [18]. Another mutation on nAChR corresponding to an arginine to threonine substitution, also known as an R81T mutation, was described as the first proven case of target-site modification leading to control failure of *Myzus persicae,* the green peach aphid, with neonicotinoids under field conditions [19]. Metabolic resistance appears to be much more common [2, 20]. Elevated expression levels of glutathione-S-transferases and esterases have been associated with resistance to *Diaphorina citri* [21] and *Aphis gossypii* [22]. Over expression of P450s monoxygenases are also frequently reported in many resistant cases. Enhanced expression level of this enzyme family has been strongly associated with resistance to neonicotinoids in *M. persicae* [23], *Bemisia tabaci* [24–26], *Trialeurodes vaporariorum* [27], *Nilaparvata lugens* [28], *Leptinotarsa decemlineata* [29] and many other pests [2]. Since the purpose of this study was to monitor for the first time the resistance level to neonicotinoids in *Anopheles* vectors, we did not consider investigating the different mechanisms involved in this resistance, which can appear as a limitation to the study. However, bioassays with synergists can provide a quick and easy basis for initial characterization of neonicotinoid resistant mosquito populations.

The sharp rise in insecticide resistance is, among other factors, favoured by the limited number of vector control insecticides which results in excessive use of the same products. Consequently, this leads to an increase in selection pressure on the targeted individuals; hence the need for new molecules with different modes of action. The time required to develop completely new compounds is extremely long so the reformulation of insecticides currently used in agriculture remains an attractive option; however, this must take into account the pressure that already exists because of the use of these molecules in agriculture. Therefore, mixture or combination formulations should be prioritized over simple formulations in order to preserve long-term efficacy and a greater impact on malaria. That is, given the assumption that the compounds could act in synergy, or that each insecticide in the mixture will be able to eliminate those individuals that are susceptible to it [30].

Beyond that, vector control needs to be rethought or re-imagined with the introduction of new mechanistic tools or interventions or strategies that go beyond mosquito nets and indoor residual spray, and that take into account local specificities. For instance, sub-Saharan African countries have intense agricultural production, and farming represents the primary source of food and/or income [31]. The use of chemicals is the principal pest control strategy commonly applied by farmers to protect their investment [31]. This can appear as an advantage for vector control. Given that swamp and wetlands used for some agricultural activities also constitute productive breeding sites for many mosquito species, agricultural pest control may become an important vector control strategy operated by farmers themselves. Indeed, the importance of larvicides in vector control programs is well known, but because its implementation involves complicated logistics and efforts to be effective, it has very often led to its abandonment. If some agricultural pesticides are reformulated and combined with selected biological larvicides, their use by the farmers could both protect their crops and control vector larvae. This approach was recently tested in Cote D’Ivoire (Chouaibou et al., in prep.) and may possess several advantages as no additional effort would be required from the farmer. In addition, savings would be made on logistics and efforts that should have been deployed by public health actors if they were to implement larviciding. Moreover, the routine nature of pesticide use in agriculture should ensure the effectiveness of the approach and it will boost traditional vector control (use of mosquito nets and indoor spraying) by attacking another link in the mosquito development chain, the larva. Such integrated pest and vector control management should be considered in depth and implemented.

## Conclusions

The findings herein provide evidence that the use of chemicals in agriculture can trigger insecticide resistance in malaria vectors. Thus, strategies to overcome the problem of resistance to insecticides in malaria vectors should take into account the major sources of resistance and be designed to delay its appearance. Data generated here could serve as a basis for the discussion of novel neonicotinoid-based vector control interventions.

## Additional files

Additional file 1: Multilingual abstracts in the five official working languages of the United Nations. (PDF 500 kb)

Additional file 2: BOTTLE TEST RESULTS SHEET of *Anopheles coluzzii* from Gagnoa. (XLS 49 kb)

Additional file 3: BOTTLE TEST RESULTS SHEET of *Anopheles coluzzii* from Vitre. (XLS 54 kb)

Additional file 4: BOTTLE TEST RESULTS SHEET of *Anopheles coluzzii* from Tiassale. (XLS 52 kb)

## Data Availability

All data generated or analysed during this study are included in this published article [and its supplementary information files].

## Abbreviations

CDC: Centers for Disease Control
DNA: Deoxyribonucleic acid
KDT: Knock down time
Min: Minute
nAChR: Nicotinic acetylcholine receptor
PCR: Polymerase chain reaction
RR: Resistance ratio
WHO: World Health Organization
