# Supplementary material for: Evidence of insecticide resistance selection in wild Anopheles coluzzii mosquitoes due to agricultural pesticide use
Source: Infect Dis Poverty. 2019 Jul 15;8:64. doi: 10.1186/s40249-019-0572-2 (PMC6631620; doi:10.1186/s40249-019-0572-2)
Supplement: Supplementary file 1 — Multilingual abstracts in the five official working languages of the United Nations. (PDF 500 kb) [file 40249_2019_572_MOESM1_ESM.pdf]

Translation of the abstract into the five official working languages of the United Nations

أدلة لمقاومة بعوض الانوفليس للمبيدات الحشرية بسبب الاستخدام الزراعي للمبيدات الحشرية.

شعيبو سيدو محمدو ، سارة سولين دي سوزا ، بهي كواديو فودجو ، ماريوس غونس زوه ، نيستور كيسي بلي ، بنيامين غيبي كودو

الملخص

خلفية: الاراضي الرطبة المستخدمة في الانشطة الزراعية تعتبر اماكن تكاثر غزيرة للعديد من انواع البعوض لذلك الإستخدام الزراعي للمبيدات الحشرية التي تستهدف حشرات أخرى قد يمكن بعوض الملاريا من مقاومة المبيدات الحشرية. الغرض من هذه الدراسه هو توضيح بعض الفجوات المعرفية بخصوص دور المواد الكيميائية الزراعية في تمكين البعوض الناقل للملاريا (الانوفليس) من مقاومة المبيدات الحشرية، وهذا بالغ الاهمية في احتواء البعوض الناقل للملاريا.

الطرق: بإستخدام علبة اختبار الس دي سي، والتي تحدد مدى قدرت المركبات الكيميائية على قتل البعوض الناقل للملاريا، وباستخدام وحدة الاحتمال اللوغارتمية، كشفنا للمرة الاولى عند مستوى مقاومة بعوض الانوفليس لالنيونيكوتينويدس، وهي مبيدات حشرية تسخدم حصريا لحماية المحاصيل الزراعية في ساحل العاج الدراسة تم إجرائها في منطقتين زراعية هي تياسل وغانيوا، ومنطقة غير زراعية هي فيتري بين شهر جون و اغسطس 2017 بإستخدام المواد المركبات الكيميائية التالية: كلوثياندين، أسيتامبريد، ايميداكلوبريد.

النتائج: البعوض في المنطقتين الزراعية، تياسل و غانيوا، كانوا مقاومين لمركب الاسيتامبريد بمعدل وفيات اقل من ٨٥ ٪ بعد ٢٤ ساعة من تعرضهم لهذا المركب الكيميائي. اما في فيتري (المنطقة الغير زراعية)، البعوض كان سريع التأثر بمركب الاسيتامبريد. في كل المناطق الثلاث، البعوض كان مقاوم لمركب الايميداكلوبريد (معدل الوفيات كان ٦٠٪ في منطقة فيتري، ٣٧٪ في تياسل، و ١٣٪ في غانيوا) و سريع التأثر بمركب الاكلوثياندين (بمعدل ١٠٠٪ وفيات). بعوض الانلوفيس مثل 100٪ من البعوض التي تم جمعها في غانيوا 86٪ في تياسل 96٪ في فيتري.

الاستنتاجات: هذه الدراسه اظهرت نتائج قوية تتمثل في ان الاستخدام الزراعي للمبيدات الحشرية قد تسبب مقاومة البعوض الناقل للملاريا لهذه المبيدات الحشرية. مقاومة البعوض للمبيدات الحشرية بسبب الاستخدام الزراعي يجب ان يوضع بعين الاعتبار عندما يتم تطوير اساليب للسيطرة على البعوض الناقل للملاريا.

Translated from English version into Arabic by Hamid Alahmari, proofread by Amira Ali, through

TRANSLATORS
WITHOUT BORDERS

农用杀虫剂对野生 *Anopheles coluzzii* 杀虫剂抗性产生的影响

Chouaïbou Seïdou Mouhamadou, Sarah Souline de Souza, Behi Kouadio Fodjo, Marius Gonse Zoh, Nestor Kesse Bli, Benjamin Guibehi Koudou

摘要

引言：某些开展农业活动的湿地会成为多种蚊虫的孳生地。因此，针对其他农业害虫的杀虫剂可能对传疟蚊媒的杀虫剂抗性选择产生影响。本研究的目的是证实农药在疟疾病媒抗药性发展中的作用，这对于病媒控制至关重要。

方法：使用美国 CDC 生物瓶杀虫剂检测法和 log-probit 分析，研究了 *Anopheles coluzzii* 对新烟碱类杀虫剂（专门用于科特迪瓦的作物保护杀虫剂）的抗药性。2017 年 6～8 月，在科特迪瓦的两个农业区域（Tiassale 和 Gagnoa）和一个非农业区域（Vitre）进行蚊虫对噻虫胺、啶虫脒和吡虫啉等三种杀虫剂的抗性检测。

结果：来自 Tiassale 和 Gagnoa（农业环境）的蚊虫种群对啶虫脒有杀虫剂抗性，处理后 24 h 死亡率< 85%。然而，在 Vitre（非农业区）蚊虫种群对啶虫脒仍较为敏感。在所有三个地区，蚊虫种群对吡虫啉均具有抗性；Vitre、Tiassale 和 Gagnoa 等 3 地蚊虫种群的死亡率分别为 60%、37%和 13%；但对噻虫胺易感（死亡率达 100%）。采自 Gagnoa 的蚊虫均为 *Anopheles coluzzii*。采自 Tiassale 和 Vitre 的蚊虫，*Anopheles coluzzii* 所占比例分别为 86%和 96%。

结论：本研究提供了强有力的证据，表明疟疾蚊媒会对农用杀虫剂产生抗性。因此，在制定媒介防制策略时应考虑由农用杀虫剂所产生的杀虫剂抗性。

Translated from English version into Chinese by Xin-Yu Feng, edited by Pin Yang

Mise en évidence d'une sélection par résistance aux insecticides chez les moustiques sauvages *Anopheles coluzzii* due à l'utilisation de pesticides agricoles

Chouaïbou Seïdou Mouhamadou, Sarah Souline de Souza, Behi Kouadio Fodjo, Marius Gonse Zoh, Nestor Kesse Bli, Benjamin Guibehi Koudou

Résumé

Contexte: Les zones humides utilisées pour certaines activités agricoles constituent des lieux de reproduction pour de nombreuses espèces de moustiques. Par conséquent, l'utilisation agricole d'insecticides qui ciblent d'autres nuisibles peut générer une résistance aux insecticides chez les moustiques vecteurs du paludisme. L'objet de cette étude, qui vise à clarifier certaines lacunes en termes de connaissances quant au rôle des produits agrochimiques dans le développement de la résistance aux insecticides chez les vecteurs du paludisme, est de la plus haute importance pour le contrôle des vecteurs.

Méthodes: A l'aide du test en bouteille des CDC et de l'analyse log-probit, nous avons étudié, pour la première fois, les niveaux de résistance des moustiques *Anopheles coluzzii* aux néonicotinoïdes, des insecticides utilisés exclusivement pour la protection des cultures en Côte d'Ivoire. Cette étude a été réalisée dans deux régions agricoles (Tiassalé et Gagnoa) et une région non agricole (Vitré) entre juin et août 2017, en utilisant de la clothianidine, de l'acétamipride et de l'imidaclopride.

Résultats: L'étude a conclu que les populations de moustiques de Tiassalé et Gagnoa (milieux agricoles) sont résistantes à l'acétamipride, le taux de mortalité étant < 85% 24 heures après l'exposition. A l'inverse, à Vitré (zone non agricole), la population de moustiques était sensible à l'acétamipride. Dans les trois localités, les populations de moustiques étaient résistantes à l'imidaclopride (les taux de mortalité étaient de 60% à Vitré, 37% à Tiassalé et 13% à Gagnoa) et totalement sensibles à la clothianidine (taux de mortalité de 100%). *An. coluzzii* représentait 100% des moustiques prélevés à Gagnoa, 86% à Tiassalé et 96% à Vitré.

Conclusions: Cette étude prouve clairement que l'utilisation agricole d'insecticides peut causer une résistance aux insecticides chez les populations vectrices du paludisme. La résistance aux insecticides générée par l'emploi de produits agrochimiques doit être prise en compte lors de l'élaboration de stratégies de contrôle des vecteurs.

Translated from English version into French by Marina Della Torre, proofread by Maëva Paoletti, through

Выборка данных по устойчивости к инсектицидам диких видов комаров вида *Anopheles coluzzii*, возникающей вследствие использования в сельском хозяйстве пестицидов

Chouaïbou Seïdou Mouhamadou, Sarah Souline de Souza, Behi Kouadio Fodjo, Marius Gonse Zoh, Nestor Kesse Bli, Benjamin Guibehi Koudou

Аннотация

История вопроса: Водно-болотные угодья, используемые в определенной сельскохозяйственной деятельности, — это благоприятные места для размножения многих видов комаров. Поэтому использование в сельском хозяйстве инсектицидов против других вредителей может привести к развитию у малярийных комаров устойчивости к инсектицидам. Целью настоящего исследования является заполнение пробелов в данных по роли, которую играют агрохимикаты в развитии у переносчиков малярии устойчивости к инсектицидам, что крайне важно при борьбе с переносчиками инфекции.

Методы: Используя диагностический экспресс-тест Центра по контролю над заболеваниями и логарифмический и пробит-анализ, мы впервые исследовали уровень устойчивости комаров вида *Anopheles coluzzii* к неоникотиноидам, инсектицидам, используемым в Кот-д'Ивуаре исключительно для защиты урожая. Исследование проводилось в двух сельскохозяйственных районах (Тиассале и Ганьоа) и в одном несельскохозяйственном районе (Витре) в течение июня и августа 2017 г. при использовании клотианидина, ацетамиприда и имидаклоприда.

Результаты: Было определено, что популяции комаров из г. Тиассале и Ганьоа (сельскохозяйственные районы) обладали устойчивостью к ацетамиприду при уровнях смертности < 85% в течение 24 часов после воздействия. Однако в г. Витре (несельскохозяйственный район) ацетамиприд был эффективен в отношении популяции комаров. Во всех трех районах популяции комаров отличались устойчивостью к имидаклоприду (уровни смертности составили 60% в г. Витре, 37% — в г. Тиассале и 13% — в г. Ганьоа) и полной восприимчивостью к клотианиду (уровень смертности — 100%). Комары вида *An. coluzzii* составили 100% комаров, собранных в г. Ганьоа, 86% — в г. Тиассале и 96% — в г. Витре.

Выводы: Результаты данного исследования свидетельствуют о том, что использование в сельском хозяйстве инсектицидов может привести к развитию у популяций переносчиков малярии устойчивости к инсектицидам. Необходимо учитывать наличие устойчивости к инсектицидам вследствие их использования в сельском хозяйстве при разработке стратегий борьбы с переносчиками инфекции.

Translated from English version into Russian by Veronika Demeshchyk, proofread by Michael Orlov, through

TRANSLATORS WITHOUT BORDERS

Evidencia de la selección de resistencia a los insecticidas en mosquitos silvestres *Anopheles coluzzii* debido al uso de pesticidas agrícolas

Chouaïbou Seïdou Mouhamadou, Sarah Souline de Souza, Behi Kouadio Fodjo, Marius Gonse Zoh, Nestor Kesse Bli y Benjamin Guibehi Koudou

Resumen

Antecedentes: Los humedales utilizados para algunas actividades agrícolas son lugares productivos para la reproducción de muchas especies de mosquitos. Por lo tanto, el uso agrícola de insecticidas dirigidos a otras plagas puede causar resistencia a los insecticidas en los mosquitos de la malaria. El objetivo de este estudio es aclarar algunos vacíos de

información sobre el papel de los agroquímicos en el desarrollo de la resistencia a los insecticidas en los vectores de la malaria que son de suma importancia para el control de vectores.

Metodología: Utilizando el bioensayo de botella de los CDC y análisis logit/probit, investigamos por primera vez los niveles de resistencia de los mosquitos *Anopheles coluzzii* a los neonicotinoides, insecticidas utilizados exclusivamente para proteger el cultivo en la Costa de Marfil. El estudio se llevó a cabo en dos áreas agrícolas (Tiassalé y Gagnoa) y en una zona no agrícola (Vitré) entre junio y agosto de 2017 usando clotianidina, acetamiprid e imidacloprid.

Resultados: Las poblaciones de mosquitos de las zonas agrícolas de Tiassalé y Gagnoa resistieron al acetamiprid con tasas de mortalidad < 85% 24 horas después de la exposición. Sin embargo, en la zona no agrícola de Vitré la población de mosquitos fue vulnerable al acetamiprid. En las tres localidades, las poblaciones de mosquitos resistieron al imidacloprid con tasas de mortalidad del 60% en Vitré, 37% en Tiassalé y 13% en Gagnoa, y fueron completamente vulnerables a la clotianidina con una tasa de mortalidad del 100%. El género *Anopheles coluzzii* representó el 100% de los mosquitos recolectados en Gagnoa, el 86% en Tiassalé y el 96% en Vitré.

Conclusiones: Este estudio proporciona pruebas sólidas de que el uso agrícola de insecticidas puede causar resistencia a los insecticidas en las poblaciones de vectores de la malaria. Durante la elaboración de estrategias de control de vectores es necesario tener en cuenta la resistencia a los insecticidas causada por el uso de agroquímicos.

Translated from English version into Spanish by Laura García Sánchez, proofread by Maria Paula Gorgone, through

TRANSLATORS
WITHOUT BORDERS
